# A Lecture on the Effects of Alcoholic Drinks on the Human System, and the Duties of Medical Men in Relation Thereto

**Published:** 1855-03

**Authors:** N. S. Davis

**Affiliations:** Prof. of Pathology and Practical Medicine, &c. &c.


THE NORTH-WESTERN

MEDICAL AND SURGICAL JOURNAL.

NEW SERIES.

VOL. IV. MARCH, 1855. NO. 3.

### ORIGINAL COMMUNICATIONS.

ART I.—*A Lecture on the Effects of Alcoholic Drinks on the Human System, and the duties of Medical Men in relation thereto.* Delivered in the lecture room of Rush Medical College on Christmas day, (Dec. 25th, 1854), in compliance with the request of the class attending the college. By N. S. DAVIS, M. D., Prof. of Pathology and Practical Medicine, &c. &c.*

GENTLEMEN :—The very short time which has elapsed since the reception of your kind invitation to address you on the present occasion, has precluded the possibility of reducing the facts and sentiments I am about to utter to writing, and hence you must be content to receive them as they occur spontaneously, without any of the pleasing influence of well studied rhetoric. The present occasion is one peculiarly appropriate for the consideration of the subject which is to engage our attention.

---

* Rush Medical College, Dec. 28, 1854.

PROF. N. S. DAVIS.

DEAR SIR—The undersigned Committee have been appointed by the Class of the Rush Medical College to return thanks to you for the able and interesting Address delivered before the Class on Christmas Day, and to request a copy of the same for publication.

H. C. CLAPP,

A. C. BUFFUM,

W. A. GORDON, } Committee,

M. R. CHADWICK,

W. H. HELLER,

A. C. BUFFUM, Secretary. Dr. J. FORD, Chairman.

MESSRS. H. C. CLAPP, AND OTHERS OF THE COMMITTEE.

The Lecture to which you allude in your kind note, this day received, was and is unwritten. But, in compliance with your request, I will furnish it for publication as soon as other pressing engagements will permit me to write it out.

With much respect, yours truly,

Chicago, Dec. 29, 1854 N. S. DAVIS.

The day which calls to mind so vividly the remembrance of "Him who spake as never man spake," and whose earthly career was spent in "doing good," may well be devoted, in part at least, to a careful consideration of those evils which are daily and hourly adding to the sum of human misery and degradation. For, though we live in a land of plenty—a land blessed above all others with all the comforts and luxuries of life, yet it requires but little examination to find poverty, misery and crime in almost every town and hamlet, from St. Johns to the Rio Grande, and from the Atlantic to the Pacific. In our own State, with its broad and fertile prairies, yielding breadstuffs enough annually to feed a population numbering millions, every Poor-House is filled with paupers and every jail with criminals. And even in this city, at this hour, there are a thousand helpless mothers and children, deprived of property, deprived of reputation, deprived of education and every right which is dear and sacred to a rational being among a free people. Some of them are in the jail as degraded convicts, some in the Bridewell for petty offences, many in the Poor-house, some are begging cold victuals or gleaning chips and sticks from the docks and ship-yards, while others still are on beds of wasting sickness, or shrinking back in terror from visions of hellish fiends who come to torment their victims before entering into the dark domain of the future. If we inquire for the cause of so much actual misery, degradation and crime in the midst of circumstances so propitious, the answer is readily found in the *millions* of gallons of alcoholic drinks annually consumed in the United States. It is these drinks, which, like the little book in the Apocalypse, though sweet in the mouth and greedily consumed by the people, yet in their vitals become more bitter than wormwood, and like an unceasing fire consume, in their turn, their property, their morals, their health, and their happiness. But it is no part of my present purpose to discuss the general subject of intemperance, or to portray the influence of intoxicating drinks on the pecuniary, social, and moral interests of society. Everything relating to these topics are already too familiar, both to yourselves and the public.—There are, however, questions still connected with this subject which require the most careful and serious investigation. The political ec nomists have long since calculated the immense pecuniary losses resulting from the use of these drinks; the moralists have portrayed in startling but truthful language their terrible influence on the moral and social condition of man; but there remains two questions which still require a careful and thorough investigation. The first relates to the influence of alcoholic drinks on the human system in a state of health, and the second to their value and efficiency as agents for the preservation and treatment of disease. From the nature of the subject, it is evident that the duty of fully elucidating these questions devolves entirely on that profession, of which you are soon to become members. It is for the purpose of assuming my share of the responsibility of this investigation that I have consented to address you on the present occasion. Aside from the direct pecuniary interest of the dealer in intoxicating drinks, the only remaining stronghold which they have on the great mass of the people, rests on their supposed efficacy as agents for strengthening or invigorating the physical system, and thereby enabling the drinker to endure fatigue and exposure to the vicissitudes of temperature, as well as to the causes of epidemic diseases with much greater impunity. Comparatively a small proportion of those who use alcoholic drinks, do so without some special excuse or supposed necessity arising out of the circumstances which surround them. One resorts to them on account of the excessive physical labor which he is called on to endure; another to increase the production of caloric in the system and thereby enable him to resist more perfectly the effects of external cold; another to prevent that relaxation and debility which accompanies extreme heat; another to prevent the injurious effects of drinking water of different localities while travelling; and still others to prevent sickness during the prevalence of unusual or epidemic diseases. Thus it is, that notwithstanding the universal acknowledgement of the pecuniary, social, and moral, evils of intoxicating drinks, yet with a large portion of the popular mind they are not only regarded as necessary, but as a kind of flaming sword capable of turning every way to guard the Eden of life. The strength of the popular faith in the invigorating and protective qualities of these drinks is shown by those provisions for the establishment of special agencies to furnish them in those states where the most stringent legal enactments have been adopted for their general suppression. To destroy this popular faith would be to take away the last stronghold of alcoholic drinks on the honest convictions of mankind, and leave them to be sustained only by the *cupidity* of the seller and the most degrading appetite of the drinker. Hence, a careful examination into the correctness of the popular ideas to which I have alluded, is of paramount importance to the interests of humanity. But, from the very nature of the subjects to be examined, none but members of our own profession have received that kind of education which renders them competent to enter upon the execution of the task. It is from a strong desire to contribute my share towards the full elucidation of this important subject, that I have consented to address you on the present occasion when so many other duties are pressing heavily upon me.

An analytical examination of the subject will readily show that the particular modes by which alcoholic drinks produce all their beneficial effects may be included under the three following heads viz:

1st. Their direct stimulant effects on the nervous system, by which the mind is excited and all the bodily functions supposed to be invigorated.

2nd. Their supposed power to sustain respiration and animal heat by furnishing carbon and hydrogen for combustion with the oxygen of the blood.

3d. Their supposed power to protect the animal system against the effects of contagions and the causes of epidemic diseases.

To a careful and rigid examination of these several propositions I shall now invite your attention. In doing this I shall endeavor to lay aside all mere moral and social considerations, and conduct the inquiry as one of purely scientific and professional interest.—The first step in the inquiry is to ascertain, if possible, the actual modus operandi, the real effects which alcohol produces primarily on the blood and elementary tissues, and secondarily on the various functions of the body. Notwithstanding the almost universal use of alcoholic drinks, during several past centuries, the information given us by writers on Materia Medica concerning their action on the physical system is meagre and unsatisfactory.

Being generally ranked at the head of the class called "diffusible stimulants," we are simply told that when taken into the stomach, they are absorbed with but little or no change, and entering the blood, excite the nervous and vascular systems, exhilerate the mental faculties, and, if taken in moderate quantities at meals, promoting digestion and the general strength of the system. In addition to this, a class of chemico-physiological writers, with Baron Liebig at their head, recognizing alcohol as a highly carbonaceous compound, and reasoning from a knowledge of chemical affinities and combinations outside of the living animal system, have claimed that it, like all the carbonaceous elements of our food and drinks, was appropriated directly to the support of respiration and animal heat by combining with the oxygen received into the blood through the lungs.

But neither the ordinary writers on Materia Medica nor the chemico-physiologists furnish us with any carefully devised and well executed series of experiments, illustrating the correctness of the views they entertain; the first seeming to derive their ideas from the *feelings* induced by alcohol, and the latter from purely theoretical notions, coupled with vague generalizations

So far as I recollect, Dr. Prout was the first to institute direct experiments for the purpose of ascertaining the effects of alcohol on the functions of the system. By collecting the expired air soon after alcoholic drinks had been taken into the system, he ascertained that it uniformly contained much less carbonic acid gas than before the drink had been taken. More recently similar experiments have been made by Sandras and Bouchardet, and seve- others, and with the same results as obtained by Dr. Prout. In the meantime, the direct observations of Beaumont on the human stomach, and the experiments of Dr. Percy and others on animals, clearly proved that the alcoholic liquids not only disappeared from the stomach in a very short time after they had been swallowed, but that they actually entered the blood, and circulated with it through all the tissues of the body with so little change in the composition or qualities of the alcohol, that its presence could be detected in the substance of the brain, liver, &c. Very recently Ducheck has made some experiments, from the results of which he claims that the alcohol on entering the blood undergoes a change, by which an ethereal substance is formed, which he calls aldehyde, and to which he attributes the exhilerating effects which follow the use of alcoholic drinks. The same observers have shown, both by experiments on animals, and by *post mortem* examinations of such as have died from the direct effects of the stronger alcoholic drinks, that the blood does not undergo the usual change in the lungs, it having been found throughout the vascular system of a dark venous color. From all these experiments and observations it is evident that the alcohol taken into the system, exerts not merely a temporary exhilerating effect on the functions of the brain and nervous systems, but also induces important changes in the blood and vital properties of all the tissues. These changes consist in a diminished exhalation of carbonic acid gas from the lungs, diminished arterialization of the blood, and diminished sensibility both organic and voluntary. The marked diminution of carbonic acid in the expired air while under the influence of alcohol, seemed to militate strongly against the doctrine that the latter underwent combustion in the blood or lungs by furnishing carbon for the oxygen inhaled. But Liebig and his followers endeavored to obviate the objection, by suggesting that the oxygen united more particularly with the hydrogen of the alcohol, thereby forming water and emitting caloric; and hence still claiming it as a supporter of combustion and animal heat. M. Ducheck, who is one of the most recent experimenters on this subject, gives us the following conclusions, viz:

"1st. Alcohol in the organism is subservient to an increased combustion, the intermediate products of which are found in the blood. 2nd. Intoxication is dependent upon the existence of aldehyde in the blood at the time. 3d. The effect of aldehyde upon the blood is that of rapid consumption of oxygen; but thereby the combustion of other substances is interrupted or rather diminished."

On these conclusions, the London Lancet for April last, has the following comment, viz:

"In these short remarks, the reader will at once perceive the most powerful inducements to abstain from alcohol. It runs away with that oxygen which he is always inspiring for the oxidation which is required in almost every process in the animal economy. We live as it were to oxidize. Almost all the changes in the body are the results of oxidation, *and yet the spirit drinker continually* checks this process of nature."—In the year 1850, I devised a series of experiments designed to test more fully the effects of alcohol on the functions of respiration, circulation and animal heat. These experiments, commenced in the winter of 1850, have been continued from time to time since. The apparatus for performing the experiments consisted of a glass tube, graduated so as to indicate the fractions of a cubic inch; a very delicately graduated thermometer; a mercury bath, and a solution of caustic potash. With these arrangements, and an intelligent assistant, in a room of equable temperature, about three hours after any food had been taken, from three to four ounces of the best brandy that could be procured was administered. But previous to administering the brandy the temperature of the body was carefully noted by inserting the bulb of the thermometer under the tongue, with the mouth closed around it for several minutes. A certain number of cubic inches of expired air was also collected in the graduated tube over mercury, and transferred from this to the bath of caustic potash, by which the amount of carbonic acid was rapidly absorbed, and its quantity indicated. Having ascertained and noted the temperature of the body, the proportion of carbonic acid in the expired air, and the frequency of the pulse before the brandy was taken, these same observations were made in precisely the same manner every thirty minutes after until three or four hours had elapsed.* In some of the experiments brandy was used as a representative of the stronger distilled liquors; and in others port wine was used, in qantities of eight ounces at a dose, to represent the fermented liquors.

The results of all my observations may be summed up as follows, viz:

1st. The most direct and obvious effect of alcohol on the human

* For the full report of these experiments see Appendix to this paper.

system is, to excite or exhilerate the functions of the brain, and increase the rapidity of the heart's action. This effect begins to be manifest within thirty minutes after the liquor is taken, and, if the dose is not repeated, perceptibly declines in from one and a half to two hours. It is the exhilerating influence of the alcohol on the brain and nerves that gives it its fascinating power over the human appetite and passions, and has induced in the popular mind the general idea that it is actually tonic, or supporting to the functions of life. The stimulant effect on the vascular system is much less than on the nervous; the pulse being increased, in my experiments, not more than from six to ten beats per minute, while its fulness and force both remained unaltered.

2d. It directly diminishes the amount of carbonic acid gas thrown out from the lungs in the expired air. This diminution begins to be apparent in less than one hour after a single dose of alcoholic liquor, and becomes more and more so until the end of two hours; when the proportion of carbonic acid begins again to increase, and at the end of three hours comes fully up to the natural proportion. The amount of diminution of carbonic acid varied in different experiments, but was well marked in all. In some instances it was diminished, for a short time, more than fifty per cent below the proportion when the experiment began.

3d. In all my experiments the temperature of the system began perceptibly to diminish at the end of one hour, and continued to do so during the two succeeding hours, the mercury generally standing *three quarters of a degree* lower at the end of three hours than when the experiment began. And at no period of time, while the effects of the alcoholic beverage remained perceptible, was there any *increase* of temperature indicated by the thermometer.

It will be seen, by examining the detailed account of the experiments given in the Appendix, that the exhilerating effects of the alcohol on the nervous tissues, and its diminution of the exhalation of carbonic acid from the lungs, are both manifested considerably before any alteration is noticed in the temperature.

The latter, however, continues to diminish for some time after the proportion of carbonic acid begins again to increase. The effect on the pulse is noticed very soon after the alcohol is taken, being first rendered more frequent, but only for an hour or hour and a half, when it comes back to its natural standard, and in some instances falls below.

From all the experiments thus far instituted, it is plainly evident that a moderate quantity of alcohol in the blood directly excites or exhilerates the functions of the brain, while it diminishes those of respiration and calorification.

I wish, gentlemen, to attract your attention particularly to the influence of the agents which form the subject of this lecture on the three great processes just named. Their influence on the functions of the brain and nerve tissue has long been known and universally acknowledged. Their effect in diminishing the proportion of carbonic acid gas exhaled from the lungs, and thereby lessening the natural process of respiration, was first directly demonstrated by Dr. Prout, and is now universally acknowledged. Their effect in diminishing the production of animal heat, was, so far as my knowledge extends, first clearly demonstrated by my own experiments, and shows an influence directly opposed to the prevalent opinion, both in and out of the medical profession. Seeing the prominent exhilerating influence of alcoholic drinks on the brain, and the patient or drinker feeling a sensation of warmth in his fauces and stomach, and soon after also a glow of heat in the face, nothing was more natural than the inference that they actually increased the temperature of the body.

No distinction, of course, was made here between the *feelings* or mere nervous sensations of the individual and his actual temperature; and hence arose the universal popular belief in the power of such drinks to increase animal heat, and consequently also in their value to man when exposed to the mere vicissitudes of temperature. When a knowledge of organic chemistry became more diffused, and alimentary substances became classified into *nitrogenous* and non-nitrogenous or carbonaceous, the former assigned as the chief agents for nourishing the tissues, and the latter for supporting respiration and animal heat, alcohol was regarded as one of the most prominent articles of the latter class, and, of course, directly calculated to support both of the last named functions.

It was thus that the theoretical inferences of the learned were made to coincide with, and apparently to corroborate those derived from the sensations of the drinker; and both were hence made to contribute largely to the continuance of those customs in society which are annually sending several thousand American citizens to untimely and dishonored graves. And yet the industrious student will not fail to perceive that these opinions, which have thus held a predominating influence over the popular mind from generation to generation, are purely hypothetical, and without a single experimental fact for their support. I am aware that some will be disposed to deny this assertion, and to allege that the experiment of taking alcoholic stimulants, when exposed to extreme cold, has been tried times without number, and with the effect to make the drinker *feel* warmer than before; and that this furnishes positive proof of the power of such stimulants to increase animal heat. That a glass of brandy will soon produce a feeling of warmth in the fauces and stomach, and sometimes in the face, I fully admit. But is this proof that the temperature of the tissues of the body is actually increased? Does not the patient, with spasmodic cholera, almost constantly complain of the heat, and plead for cold water—ice cold water—even when his whole body, and the very breath from his lungs, are cold as death itself? Are his feelings, under such circumstances, any index of the actual temperature? Certainly not. Again, a patient, under the influence of chloroform or ether, revels in a world of dreams, and perhaps pleasing fancies, all unconscious of the fact that his limbs are being severed from his body, or his flesh extensively lascerated in the extirpation of morbid growths; but is it any proof that the limb is not severed because the patient did not feel each stroke of the surgeon's knife?

So, too, when alcoholic drinks are taken into the stomach, a sensation of warmth is felt in the stomach and pharynx, and, as the fluid enters the blood, and through it, comes in contact with the brain and nerve tissues, its direct exciting effect is accompanied by a sensation of heat in the face, and a diminished consciousness of the presence of a material body.

But this *feeling* of heat in the face, pharynx, and stomach, under the influence of alcoholic drinks, is no more evidence of an actual increase in the temperature of the body than is the absence of pain, on the part of the patient, under the influence of chloroform, evidence that the surgeon's knife has not severed the tissues. In both instances the brain and nervous system are under the influence of an agent which alters their sensibility, and renders the individual incapable of judging correctly concerning the impression of external agents upon him.

Hence the alcoholic drinks, instead of protecting the individual against the effects of external cold, only render him less conscious of the existence of such cold, and thereby often induce him recklessly to remain exposed until fatal effects are produced. So true is this that nineteen twentieths of all those who are frozen to death in Christendom, are so while directly under the benumbing influence of alcoholic drinks, taken under the delusive idea of "keeping them warm."

I am fully assured that no fact in the whole science of medicine is susceptible of a clearer demonstration than that alcoholic liquors, taken into the human system, exert a direct and important influence over the three great functions of innervation, respiration, and calorification.

The first it temporarily excites, accompanied by a still more temporary excitement of the circulation, while the two latter are directly and positively diminished, accompanied ultimately also by a diminution of the *first*. Let us now examine a little more in detail the *modus operandi* by which these results are produced. Dr. Henry Parker, of this city, a former pupil of mine, in a prize essay presented to the Illinois State Medical Society, thus sums up the mode by which alcoholic drinks produce the effects I have described, viz.,

"1st. By their great affinity for, and absorption of the oxygen of the blood, thereby interfering with its agency in the formation of plastic material, and impairing the organizability of those compounds designed for nutrition and reproduction.

"2d. By preventing or retarding that vital change—*the conversion of venous blood into arterial*, and diminishing the functional activity of the secreting and excreting structures generally, thus causing a retention and accumulation in the blood of effete and excrementitious compounds.

"3d. By retarding capillary circulation and the metamorphosis of the tissues."

That alcohol possesses an affinity for oxygen, and readily enters into combination with it under favorable circumstances, is well known. M. Bouchardet, from his experiments, first supposed that the alcohol meeting the free oxygen received into the blood through the lungs, combined with it in such proportion as resulted in the formation of acetic acid and the disappearance of the alcohol. M. Duscheck more recently contended that the latter, by its first union with the oxygen, resulted in the formation of aldehyde, which, however, maintains but a temporary existence, being, by further combination with oxygen, converted into acetic, oxalic, formic, and carbonic acids. It was this supposed union of alcohol with the oxygen of the blood that led Liebig and his followers to regard such union as a *combustion*, and consequently the alcohol as a strong supporter of animal heat. But, admitting that the union takes place, (of which we are not quite certain), their conclusion by no means necessarily follows. No fact in science is better established than that the free oxygen in the blood is the great vivifying or life-sustaining agent, which, by its presence, maintains the susceptibility and action of all the tissues of the body, and, by its chemical combinations, facilitates both the nutrition and metamorphosis of the tissues. Neither is any fact better established than that the natural temperature of the body is sustained by these same processes. Hence if it were true, as represented by Liebig, Duscheck, &c., that the alcohol entered into direct combination with the oxygen of the blood, and even developed sensible caloric by so doing, such development would be more than counterbalanced by the diminished quantity resulting from these organic processes, which are directly impaired by the diversion of oxygen from its natnral and healthy offices in the economy. Whether or not the alcohol taken into the system actually enters into combination with the oxygen of the blood, thereby diverting it from its natural affinities and uses, it is certain that its presence in the blood causes a rapid accumulation of carbonic acid in that fluid, and a decided diminution of the change from a venous to an arterial hue as it passes through the pulmonary organs. Thus, Dr W. B. Carpenter, the author of a large and standard work on Human Physiology, says that M. Buchardet found, "when alcohol is introduced into the system in excess, the blood in the arteries presents the aspect of venous blood, showing that it has been prevented from undergoing the proper oxygenating process." The same dark venous color of the blood in the arteries was observed by Percy, and many others, both in experiments on animals, and in post mortem examinations of persons who had died while strongly under the influence of the stronger alcoholic drinks. Indeed, plain evidence of this want of proper change of the blood from venous to arterial color may be observed in the dingy and leaden hue of the countenance, the purple color of the lips and nails, and the slow circulation of the blood in the capillaries of the surface, of persons in a state of profound intoxication. The dark color of the arterial blood, together with the diminished elimination of carbonic acid gas from the lungs, while under the influence of alcoholic drinks, demonstrates, beyond all cavil or doubt, the depressing influence of these drinks on the function of respiration, and the vital changes which accompany it.

This effect on the respiratory function is not fully explained on the supposition of Liebig and Duscheck, that the alcohol unites directly with the oxygen of the blood, thereby forming new compounds, consisting chiefly of acetic and carbonic acids and water. It might explain the increase of carbon in the blood, and the diminished arterial color, but does not furnish a satisfactory reason why the amount of carbonic acid thrown off with the expired air so rapidly diminishes. Its mere increased accumulation or formation in the blood should lead rather to an increased elimination, which certainly does not occur It is well known that alcohol possesses a strong affinity for animal membranes and albuminous tissues generally; not merely permeating them with readiness but entering into actual combination with them in such a manner that no ordinary washing or masceration will fully remove it, and at the same time so altering their structure as to make them appear more dense and corrugated. That the same effect is produced on *living* animal membranes and tissues is rendered extremely probable by many experiments. Thus, if the tail of a tadpole or leech be immersed in alcohol only a few seconds, it becomes stiff as far as the immersion extends, and remains incapable of regaining either its flexibility or excitability. The same rigidity is produced by immersing frogs and puppies; but with them the effect slowly subsides if the immersion is not too long continued. Humboldt found the direct application of alcohol to the larger nervous cords of the frog to produce, first a very temporary excitement, followed by an entire loss of action and *excitability.*

These facts, taken in connection with the rapid diminution of exhaled carbonic acid, and the positive diminution of the temperature, as proved by my own experiments, leave very little doubt but that the alcohol exerts an influence, immediate and direct, on the tissues of the body, from the strong affinity it possesses for the albumen, which enters so largely into their composition.

The immediate effect of this affinity is to arrest or retard all the more minute molecular changes which are constantly taking place in the healthy state of the living tissues. So long as it maintains its existence and direct affinity for the tissues, its presence excites or exhilerates the brain and nerves, accompanied by a diminished consciousness of outward objects and impressions. These are the primary effects of alcohol on the system ; and while they continue they constitute the period of intoxication or apparent excitement. But its contact with the oxygen of the blood at a temperature of 97° or 98° F., soon induces a change in its composition, and leads to the formation of those compounds pointed out by Liebig and Duscheck. This constitutes the second period in the action of alcoholic drinks on the system, and is characterized by a rapid diminution of the cerebral exhileration, and the final occurrence of mental depression, muscular weakness, loss of appetite, and a return of the full proportion of carbonic acid in the expired air. Such are the effects of a single moderate dose of alcoholic drink. But when the amount taken is very large, or more moderate doses are repeated at short intervals, the obstruction to the elimination of the carbonic acid from the lungs is more protracted, which causes it to accumulate in the blood to such an extent that the susceptibility of the nerve structures is at length overcome by the venous condition of the whole mass of the circulating fluid, the brain consequently no longer responds to the exhilerating influence of the alcohol, and the individual becomes stupid, unconscious, and lethargic, with slow breathing, livid lips, feeble capillary circulation, and entire muscular prostration.

He is said to be "*dead drunk.*" Let the process be carried one step farther, and the blood will be altered to such an extent that the susceptibiltty of the medulla oblangata will also fail, and life itself will be extinct. If the supply, however, is only sufficient to induce stupid drunkenness, the patient remains in a half comatose or lethargic condition several hours, during which time the alcohol becomes decomposed to such an extent that the air cells and pulmonary tissue permit again the absorption of oxygen and the elimination of carbonic acid, by which the blood is rapidly freed of its excess of effete matter, the susceptibility of the brain gradually returns, and the patient wakes from his lethargy with feelings of depression, timidity, and muscular weakness, that last for some time longer. Such are the physical effects of alcoholic drinks on the human system; and they may be summed up in the following concise and simple propositions, viz.:

1st. They are rapidly absorbed from the stomach, and enter with the mass of the blood directly into contact with all the tissues of the body.

2d. By their presence in, and the affinity of the alcohol for the tissues, they induce a peculiar exhileration of the cerebral and nervous functions, while they so alter the membraneous structures, including the air cells, that the exchange of oxygen and carbonic acid gases through the latter is much diminished, and all the organic actions so retarded as to induce a perceptible diminution of the temperature of the body.

3d. The alcohol itself, by continued contact with the oxygen and other constituents of the blood, gradually undergoes decomposition, giving rise to the formation of acetic and carbonic acids and water; which, added to the previous interference with the respiratory process, causes the blood to become loaded with effete matter, more venous than natural, and incapable of maintaining the susceptibility and tone of the nervous and muscular structures.

With this, perhaps tedious, examination of the modus operandi of alcoholic drinks, you are prepared, gentlemen, to see clearly how far their use is calculated to produce those beneficial effects which have been so generally ascribed to them by a large portion of the people. You can see, with the clearness of demonstration, that, instead of furnishing fuel for *combustion*, and thereby supporting respiration, and increasing animal heat, they primarily depress both these important functions, and directly retard the change from venous to arterial blood. Instead of increasing innervation, and *invigorating* the various functions of the body, we see them merely exerting a peculiar exhilerating influence upon the brain, by which the natural consciousness and susceptibility to impressions are impaired, and the muscular actions rendered feeble and vascilating. In this perverted susceptibility of the nervous structures lies the power of alcohol to deceive the popular mind. With a glass of brandy in a man's blood, he quickly imagines himself as rich as Cresus, though actually clothed in rags, and as strong as Sampson, though really trembling in every muscle, and his knees smiting each other like those of a disheartened Philistine. So true are the words of the good book, when it says, "*Wine is a mocker, and strong drink is raging, and whosoever is deceived thereby is not wise.*" You can judge, too, how far an agent or agents which thus retard one of the most important excretory functions in the system, diminish the change from venous to arterial blood, and cause in the latter fluid an undue accumulation of effete or waste matter, and an ultimate depression of all the organic actions, is calculated to counteract the influence of morbific agents in the production of disease.

You readily perceive that all its effects, both direct and indirect, characterize it as one of the most efficient predisposing causes favoring the development of disease in the system, and rendering it more fatal when it does occur. The only exceptions to its deleterious influences are, first, when during the actual progress of disease there is danger from the direct *failure* of action in the brain and nervous centres, as is sometimes the case in the advanced stages of typhus fevers, when the direct exhilerating influence of the alcohol may be made available for temporarily sustaining the nerve sensibility; and second, when the process of disintegration or waste of tissues takes place much too rapid, with an active state of the excretory organs, as is sometimes the case in the advanced stage of phthisis, when the power of alcohol to retard organic changes may be rendered beneficial, to a limited extent, in retarding the process of waste.

Such, gentlemen, are my views of the action of alcoholic drinks on the human system; but some may be disposed to say, in reply that those drinks have been in common use for many generations, that the popular ideas in regard to their beneficial effects in the various relations of life are founded on direct experience, and that such experience is entitled to more confidence than all the deductions of science.

I admit that these beverages have been in common use for many generations; and so has tobacco, and many other things, that neither prolong human life nor prevent disease. But what is the result of all this long experience in the use of intoxicating drinks? Has it all furnished us with any clear proof that they are capable of increasing man's power to endure either cold or fatigue, or to resist more successfully the causes of disease and pestilence? I answer no, not any—not even a shadow of such proof. During the last fifteen years I have carefully and diligently searched the dark record of alcohol's doings, on the land and on the sea, in the frozen regions of the North, and on the sultry plains of the South, in the city and in the country, on the farm, in the workshop, in the manufactory, and on ship-board; but nowhere have I found the least proof of the beneficial effects alleged. And I boldly challenge the world to furnish such evidence. On the contrary, I have found abundant evidence to corroborate the inferences drawn from the scientific part of our inquiry. For instance, if we follow the earlier expeditions in search of a north-west passage amidst the icebergs of the Northern ocean, we are told by every witness that the use of intoxicating drinks actually diminished the power to sustain severe and protracted cold.

If we examine the records of injured and disabled vessels at sea, where directly exposed to cold and sleet, it becomes necessary to keep the pumps manned day and night, we shall find several instances in which direct experience absolutely compelled an entire abandonment of all alcoholic beverages, while we shall not find one of a reverse character. On the other hand, the records of the harvest field, the brick-yards, and all other places where severe and protracted manual labor is endured, show that more labor is actually performed in a given time, and less sickness endured by those who wholly abstain from all intoxicating drinks, than by an equal number of those who indulge in their use.* In regard to their influence as preventives of disease, experience furnishes us still more abundant data.

A few years since an examination of the Report of the Register General of the British army in the East Indies, showed that there were in the different regiments occupying that insalubrious country a considerable number of *teetotallers*, and of their number only an average of little more than *three* per cent had been daily on the sick list during the year, while of those in the same regiments, subjected to the same duties, who took their regular rations of alcoholic drinks, an average of more than *ten* per cent were daily on the sick list. Observations in the West Indies have shown a similar result. What is thus true of diseases generally is still more apparent during the prevalence of severe and fatal epidemics. The records of every cholera pestilence, both in hospital and in private practice, show that those individuals and classes who are most addicted to the use of intoxicating drinks, furnish by far the larger proportion of victims to this scourge. This is not only true in relation to those who drink to excess, but also in regard to all classes who drink, however moderately. I have myself toiled through five seasons of epidemic cholera without being absent from my post of duty twenty-four hours at a time. I have passed from one sick bed to another in the midst of cholera pestilence for ten or twelve successive days and nights without resting *two hours* out of twenty-four; and so thoroughly am I convinced that alcoholic drinks, instead of preventing, strongly predispose the system

* For interesting facts on this subject, see Appendix, Note B.

to an attack, that I would as soon attempt to extinguish fire by pouring oil on it, as to take a glass a day of alcoholic drinks while exposed to epidemic influences. If there is any time when all the machinery of life should be left untrammeled and free, and especially when the blood should be well arterialized, and the excretory organs active, it is when we are surrounded by special causes of disease, and perhaps the very atmosphere we breathe loaded with noxious materials.. For the same reason the very common practice of mixing brandy with water when travelling through districts of country to which we are not accustomed, is far more frequently the cause than the preventive of evil effects.

I will mention but one other fact strongly illustrating the influence of alcoholic drinks on human *health* and *longevity*. It is well known that Life Insurance Companies have been established in most of the enlightened nations of the earth.

To afford them a safe principle of action, much care has been taken to ascertain the average duration of human life, and the influence of certain agents thereon. And so fully has it been shown that the use of alcoholic drinks materially shortens the average duration of life, and thereby adds to the risk of insurance, that companies have been established, both in this country and in England, for granting policies only to those who abstain wholly from these drinks, and that at a lower rate of premium than is required by companies which make no distinction in regard to the moderate use of intoxicating drinks.

Some of these *temperance* insurance companies have now been in active operation several years, and so satisfactory have been the results, that one of the best life insurance companies in New England has recently opened a special *temperance department*, with rates of life insurance 25 per cent below the ordinary rates of other companies doing a promiscuous business, and the same degree below their own rates in the other department

I might thus direct your attention to every department of human industry, and every relation of human life, and in all we should find the actual results of experience with the use of intoxicating drinks in consonance with the inferences to be drawn from my views of their physiological action on the system. And these inferences are, that they debase the intellectual and moral faculties, deteriorate the quality of the blood, disorder most of the functions, diminish man's power of physical endurance, and shorten the duration of human life; effects all of which are generally induced in proportion to the quantity and quality of the liquor used. If these views are correct, an immense responsibility rests upon that profession of which you, gentlemen, are soon to become members. This responsibility arises from the fact that our profession is the only one well fitted, by its education, to understand the action of alcohol on the physical condition of man, in health and disease. The practising physician also holds a closer relation, and gains a freer access to the elements of society, as represented in the family circle and the individual mind. No age, no sex, and no class of poople escape his contact and influence. He goes, in many instances, where the philosopher, the moralist and the divine can gain no access; and from the very fact that he is a physician, his supposed acquaintance with all that relates to health and the action of exterior agents on man, renders both his precepts and example doubly influential. But just in proportion as the members of our profession possess exclusive and superior knowledge concerning the influence of physical agents on health and happiness—in proportion to the freedom and extent of their access to the minds and hearts of all classes, in the same proportion is their responsibility increased. I fear, gentlemen, that few among us realize the extent of this responsibility, or even the potency of their own example. To illustrate the latter I will relate an incident which occurred during the past summer, when cholera was prevailing severely in this city. It was about the middle of July, when Mr. A., of the village of O., in the interior of the State, came into the city to transact some business. While here, like too many others, he stepped into one of the most fashionable saloons of the city, and soon a well dressed gentleman passed in, and up to the bar, and called for a glass of brandy. As he entered, several citizens exclaimed, in a salutary tone, "Well, Dr. B., how is the cholera to-day?" "Pretty bad," answered the Doctor, as he took his glass; "about thirty deaths during the last twenty-four hours;" and he hastily retired.

Our friend from the country tarried a few minutes, and soon came Dr. C., who, in answer to inquiries, gave about the same information as Dr. B., took a glass of brandy with some friends, and also retired. Mr. A., in his travels about the city, had already met several funeral processions, and as he stepped out of the saloon another was passing, which, with the conversation to which he had just listened, created an anxiety to get out of the city as soon as possible.

Now Mr. A. was strongly inclined, though not positively pledged, to practice total abstinence from all intoxicating drinks. But seeing, in the course of a few minutes, several well dressed citizens, and two apparently prominent physicians, freely quaff from the intoxicating cup, mingled with remarks in regard to its necessity in such sickly seasons, he was induced, rather reluctantly, to call for a glass himself before starting on his homeward journey.

On his arrival, "what news from the city?" was of course the first question asked by his neighbors and friends. "Not much news," he replied; "rather dull times in everything but the saloons and coffin ware-rooms, for the cholera is raging there badly. I saw two physicians who said there had been more than 30 deaths from the disease during the preceding day, and the streets were full of funeral processions." "I wonder," said neighbor D., "if there is not some preventive; something that one could take to lessen the liability to an attack." "I presume there is," said Mr. A., "for while in the city I heard those who were talking about it say that a little *pure brandy* was an excellent thing to prevent the relaxing influence of the warm weather, and both of the physicians I saw took brandy themselves." This hint derived from Mr. A., backed as it was by the *example* of two city physicians, was sufficient to start a large number after the proposed remedy.

And it was an actual fact that in less than a week the village drug store was completely exhausted of its stock of brandy, it having all been bottled and labelled "Pure Brandy," and sold at the highest prices.

This anecdote is not only literally true in regard to the village or town referred to, but affords a good example of what may be observed in every section of the country wherever epidemics or rumors of epidemics excite the fears of the people.

And the great day of final judgment can alone reveal the vast amount of drunkenness, vice, and death which have thus directly and indirectly resulted from the idea that intoxicating drinks were calculated to invigorate the system, and protect it from attacks of disease. It is, indeed, this idea, still deeply rooted in the popular mind, and countenanced by too many physicians, that now constitutes the great barrier in the way of totally exterminating both the traffic in, and the use of these drinks throughout the several States of our Union. Hence it is that legislators, though anxious to dry up the streams of pauperism, vice, and crime that flow from the dram-shops by just and stringent laws, are yet constrained to provide for special town *agencies* to sell for sacramental and medicinal purposes.

Now every such *agency* is a monument of disgrace to the medical profession of the last half of the nineteenth century.

For what do we need the use of intoxicating drinks in medicine?

Is it for the hops in the beer, the acids in the various wines, the juniper in the gin, or the various poisonous adulterations with which nineteen-twentieths of the intoxicating liquors of this country are mixed? Certainly it is not for any of these, but simply for the great exhilerating agent, alcohol, which enters as a common ingredient into them all. Then why not, as a profession, use *alcohol* as such, and at once sever all connection with that whole group of intoxicating beverages which have so long scourged the human race? There is not a single medicinal effect to be obtained either by the fermented or distilled spirits that cannot be obtained with equal certainty by any intelligent physician, with alcohol diluted and combined with other substances to suit the particular case; neither is there a single useful purpose, either in the arts or commerce, to which the alcoholic drinks can be applied, that cannot be served equally well with alcohol, more or less diluted. Hence there is no more necessity for establishing special *town agencies* to sell intoxicating drinks, than there is to sell opium or castor oil. The truth is, that alcohol, as an article of commerce, should be manufactured and sold in the same manner and under the same regulations as paints, oils, varnishes, and drugs generally; and neither it nor any of its diluted compounds, in the form of fermented or distilled spirits, should be permitted to be kept, sold, or used as drinks, any more than we should permit chloroform, ether, or opium to be kept and used for the same purpose.

But, gentlemen, I am trespassing upon your time and patience too far, and will hasten these observations to a close. Thus far I have confined my remarks to the influence of alcoholic drinks on the physical man, and the relations which the profession hold to their common and destructive use. If the views I have given you in relation to their physical action on the functions of the animal economy are correct, you will see the fallacy of that often repeated remark, that the evils resulting from intoxicating drinks, arise from their *abuse*, and not necessarily from their *use*.

You will see clearly that the very nature of their action on a healthy condition of the system is injurious, and therefore that their use in any degree, as beverages, is an *abuse*. It is one of the highest and noblest aims of the profession to which we belong, to *prevent* disease and suffering, as well as to alleviate and cure it. Hence we point with just pride to the discovery of vaccination by Jenner, and all other means by which we have been enabled to prevent disease, and add to the average duration of human life. But could we, as a profession, remove those popular errors which now sustain and propagate the use of alcoholic drinks, and thereby secure their entire banishment from Christendom, we should confer on the human race a boon, in comparison with which the great discovery of Jenner would sink into insignificance.

One thought more, gentlemen, and I have done. Most of you are just entering upon the active, influential period of life, in connection with a profession whose members are exposed, on the one hand, to severe labor and strong temptations, and on the other, sustained by the most attractive fields of science, and the noblest motives that can actuate the human mind. There are hence two paths open before you, the one leading close by the stagnant pools of indolence and self-indulgence, closely beset by the allurements of vice and pecuniary gain, bnt ending in individual degradation and professional disgrace. The other leads directly over the rugged hill of science, on the summit of which ever bloom flowers of purity and affection, and down whose farther side courses the limpid streams of knowledge. At every turn of this path are seen signs of industry, perseverance, and self-denial, while at its terminus the eye rests on the traveller with whitened locks, and a serene, happy countenance, holding in one hand a crown, richly decked with tokens of affection and gratitude, mingled with testimonials of merits acquired and benefits conferred; and in the other, a simple viol of oil, emblematic of the healing influence exerted by his life on both the moral and physical maladies of his race. It is for you to choose, gentlemen, which of these paths you will travel.

The entrance to both is immediately before you, and only a step or two is required to place you in either. A single bad habit may be the fatal step which will lead you into the first, while it will require an aggregation of many good ones to enable you to travel successfully the latter. It is, therefore, of the utmost importance that, in the commencement of active life, the outward actions should be correct, and also that those actions should spring directly from the purest and most elevated motives. It is not more certain that the simple germ enclosed in the acorn, when exposed to a moist and genial soil, will grow until its gnarled trunk and spreading branches defy alike the sunshine and the storm, than that a single unholy motive or vicious habit, knowingly indulged, will extend its poison roots into every crevice of the soul, and its odious branches into all the outward manifestations of character. We too often forget that the dew-drops which glitter in the rays of the morning sun, when aggregated, form the vast heaving ocean; or that the harmless sparks of the electric machine, when aggregated, make the lurid lightning that illumines the heavens with its flash, and makes the earth tremble with its shock. But these are no more true than that the little acts and habits of each day, when aggregated, make up the individual character, nay the whole of individual life. Hence, in forming an elevated character—a pure, noble, and useful life, all the elements and items of which it is composed, in the form of daily habits, motives, and actions, must be equally pure and elevated. This is not more important in the relations of man to his fellow, than in his relation to himself. For of all the forms of servitude none are so abject, so degraded and loathsome, as that which brings the whole man in subjection to the lower and more vicious propensities and passions of his own nature. On the other hand, there are none so noble and truly free as those whose characters are based on *temperance*, *virtue*, and *truth*.

### APPENDIX.

### A.

### EFFECTS OF INTOXICATING DRINKS ON THE PHYSICAL CONDITION OF MAN.

*Experiment* 1. December, 1850, 9 o'clock P.M., three and a half hours after supper, the temperature of the body, as indicated by a delicately graduated thermometer, under the tongue, was 95 ° F., and the proportion of carbonic acid in the expired air 1 in 13. The pulse was 80 per minute, which was about the normal standard for the individual. Four ounces of strong brandy were now taken. At the end of 45 minutes the pulse was increased to 90 per minute; the temperature remained unaltered; but the proportion of carbonic acid gas in the expired air was diminished to 1 in 15. At the end of one hour the pulse had fallen to 85 per minute, the temperature still unaltered, and the proportion of carbonic acid gas still further diminished. At the end of two hours the pulse had returned to 80 per minute, the thermometer indicated the temperature at 94° 8 F., while the proportion of cabonic acid expired was perceptibly increased. At the end of three hours, the temperature had fallen perceptibly below 94° 5 F., and the proportion of carbonic acid gas exhaled had increased again to 1 part in 12, 5.

*Experiment* 2. On the 18th of October, 1852, after remaining quiet two hours in a room, the temperature of which was kept steady at 70 ° Fahrenheit, at 8¾ o'clock P.M., my temperature, as indicated by a delicately graduated thermometer, the bulb of which was inserted under the tongue, was 98¼ ° F., the pulse 76

per minute, and respiration 17. I took at once three ounces of brandy, mixed with water. *Thirty* minutes after the brandy was taken, I felt an unusual dryness in the mouth and fauces, a feeling of exhileration in the head, with a slight general feeling of numbness throughout the whole system, and a sensation of increased heat, especially in the stomach and face; the thermometer, applied in the same manner as before, indicated the temperature at 98 ° F., the pulse 84, and respiration 17. *Sixty* minutes after taking the brandy, all the feelings just mentioned were much increased, the sense of exhileration in the head amounting to decided giddiness or feeling of intoxtcation; the temperature was $97\frac{3}{4}$ ° F., pulse 77, and respiration 17. *One hundred and twenty* minutes after taking the brandy, all the feelings before described had begun slightly to diminish; the temperature was $97\frac{1}{2}$ F., the pulse 75, and respiration 16.

In thirty minutes more the temperature was found the same, the pulse 72, and respiration 16, with a further diminution of unnatural feelings. The exhileration in the head, had now become converted into a sensation of tightness and dull pain. At each time of noting the temperature, pulse &c., a given quantity of *expired* air was collected, and the quantity of carbonic acid in it ascertained. During the first hour after taking the brandy, the relative proportion of carbonic acid exhaled, underwent a rapid and marked diminution. At the end of two and a half hours, it was fully equal to the proportion before the experiment began. In all the examinations of temperature, the bulb of the thermometer was inserted under the tongue, with the mouth closed around it for ten minutes, and the height of the mercury noted by a careful assistant before its removal.

*Experiment* 3.—Nov. 8., 1852, 9 o'clock P. M., in the same room, with the temperature, regulated in the same *manner* as in experiment number 2. Three hours after a light supper, my temperature under the tongue was $97\frac{3}{4}$ F., pulse 72, and respiration 18 per minnte. I drank at once 8 *ounces of good* old Port Wine. In *thirty* minutes after drinking the wine, my temperature was $97\frac{5}{8}$ F., pulse 78, respiration 19.

Ten o'clock P. M., one hour after taking the wine, feel much exhilaration of mind and brain, eyes hot, mouth and fauces dry, a sense of numbness in the hands, face and throat; muscular weakness or a feeling as though I should stagger without an effort to prevent it. Temperature $97\frac{3}{8}$° F., pulse 77, and respiration 19.

Eleven o'clock, two hours after the wine, same unnatural feelings continue, with more approach towards head-ache and a sense of dulness and tightness above the ears. Temperature $97\frac{1}{8}$° F., pulse, 73, and respiration 19.

Twelve o'clock, three hours after the wine, mouth less dry, but a dry bad feeling in the throat, less exhileration and more pain, with heaviness in the head, and some general feeling of lassitude. Temperature 97° F., pulse 68, and respiration 17.

The effect on the relative proportion of carbonic acid in the expired air was the same in this as in the preceding experiment.

In both experiments other qualities of the pulse besides mere frequency, were carefully noted, and the gentleman who assisted me thought that its fulness and firmness was diminished in pretty close proportion to its increase in frequency. The execretory evacuations were also noted carefully during the 24 hours following each experiment, but no change was observable, except a slight diminution in the quantity, with a deeper red color, of the urine. Its specific gravity and chemical composition were not examined.

The foregoing experiments have been repeated, and varied, but without changing the results; and it is unnecessary to detail them all.

---

B.

It was my purpose to collect here some interesting statistics alluded to in the accompanying address. But the pressing nature of other duties has prevented. Those readers who either doub the statements in the address, or who wish the statistics more in detail, will find abundance of them in the last volume of the British and Foreign Medical Review, before it was united with the Medico-Chirurgical Review, and also in Dr. Carpenter's work on the effects of alcoholic drinks on the human system; which last may be found in all our book-stores.

